# Pluripotent stem cell-derived CAR-macrophage cells with antigen-dependent anti-cancer cell functions

**DOI:** 10.1186/s13045-020-00983-2

**Published:** 2020-11-11

**Authors:** Li Zhang, Lin Tian, Xiaoyang Dai, Hua Yu, Jiajia Wang, Anhua Lei, Mengmeng Zhu, Jianpo Xu, Wei Zhao, Yuqing Zhu, Zhen Sun, Hao Zhang, Yongxian Hu, Yanlin Wang, Yuming Xu, George M. Church, He Huang, Qinjie Weng, Jin Zhang

**Affiliations:** 1 Center for Stem Cell and Regenerative Medicine, Department of Basic Medical Sciences, and The First Affiliated Hospital, Zhejiang University School of Medicine, Hangzhou, 310058 China; 2 Institute of Hematology, Zhejiang University, Hangzhou, 310058 China; 3 Zhejiang Province Key Laboratory of Anti-Cancer Drug Research, Center for Drug Safety Evaluation and Research, Institute of Pharmacology and Toxicology, College of Pharmaceutical Sciences, Zhejiang University, Hangzhou, 310058 China; 4 The First Affiliated Hospital, Zhejiang University School of Medicine, Hangzhou, 310000 China; 5 Department of Neurology, The First Affiliated Hospital of Zhengzhou University, Zhengzhou, 450052 China; 6 Department of Genetics and Wyss Institute for Biologically Inspired Engineering, Harvard Medical School, Boston, MA 02115 USA; 7 Zhejiang Laboratory for Systems and Precision Medicine, Zhejiang University Medical Center, 1369 West Wenyi Road, Hangzhou, 311121 China

**Keywords:** Chimera antigen receptor (CAR), Induced pluripotent stem cells (iPSC)-derived macrophage cells (iMac), Antigen-dependent activation, Anti-cancer cell functions

## Abstract

The Chimera antigen receptor (CAR)-T cell therapy has gained great success in the clinic. However, there are still major challenges for its wider applications in a variety of cancer types including lack of effectiveness due to the highly complex tumor microenvironment, and the forbiddingly high cost due to the personalized manufacturing procedures. In order to overcome these hurdles, numerous efforts have been spent focusing on optimizing Chimera antigen receptors, engineering and improving T cell capacity, exploiting features of subsets of T cell or NK cells, or making off-the-shelf universal cells. Here, we developed induced pluripotent stem cells (iPSCs)-derived, CAR-expressing macrophage cells (CAR-iMac). CAR expression confers antigen-dependent macrophage functions such as expression and secretion of cytokines, polarization toward the pro-inflammatory/anti-tumor state, enhanced phagocytosis of tumor cells, and in vivo anticancer cell activity. This technology platform for the first time provides an unlimited source of iPSC-derived engineered CAR-macrophage cells which could be utilized to eliminate cancer cells.

## To the Editor,

Recently, CAR-iPSC-differentiated CAR-expressing T cells and NK cells have been reported to have potent cytotoxic activity against cancer cells, and they represent a new family of engineered stem cell-derived immune cells for CAR therapies [1, 2]. Myeloid cells such as macrophages have been utilized as a type of effector cells to combat cancer cells by means of their phagocytosis function [3, 4]. However, immortalized macrophage cell lines are not applicable to clinical settings, and bone marrow or PBMC-derived primary macrophages are not efficiently engineered, thus leaving iPSC-derived macrophage cells as a great source for myeloid cell-based immunotherapy. Upon challenge with antigen-expressing cancer cells, CAR-expressing iPSC-induced macrophage (CAR-iMac) cells showed antigen-dependent macrophage functions. Expression of a CAR targeting tumor-associated antigen conferred CAR-iMac cells in vitro and in vivo anti-tumor effects.

**Fig. 1:**
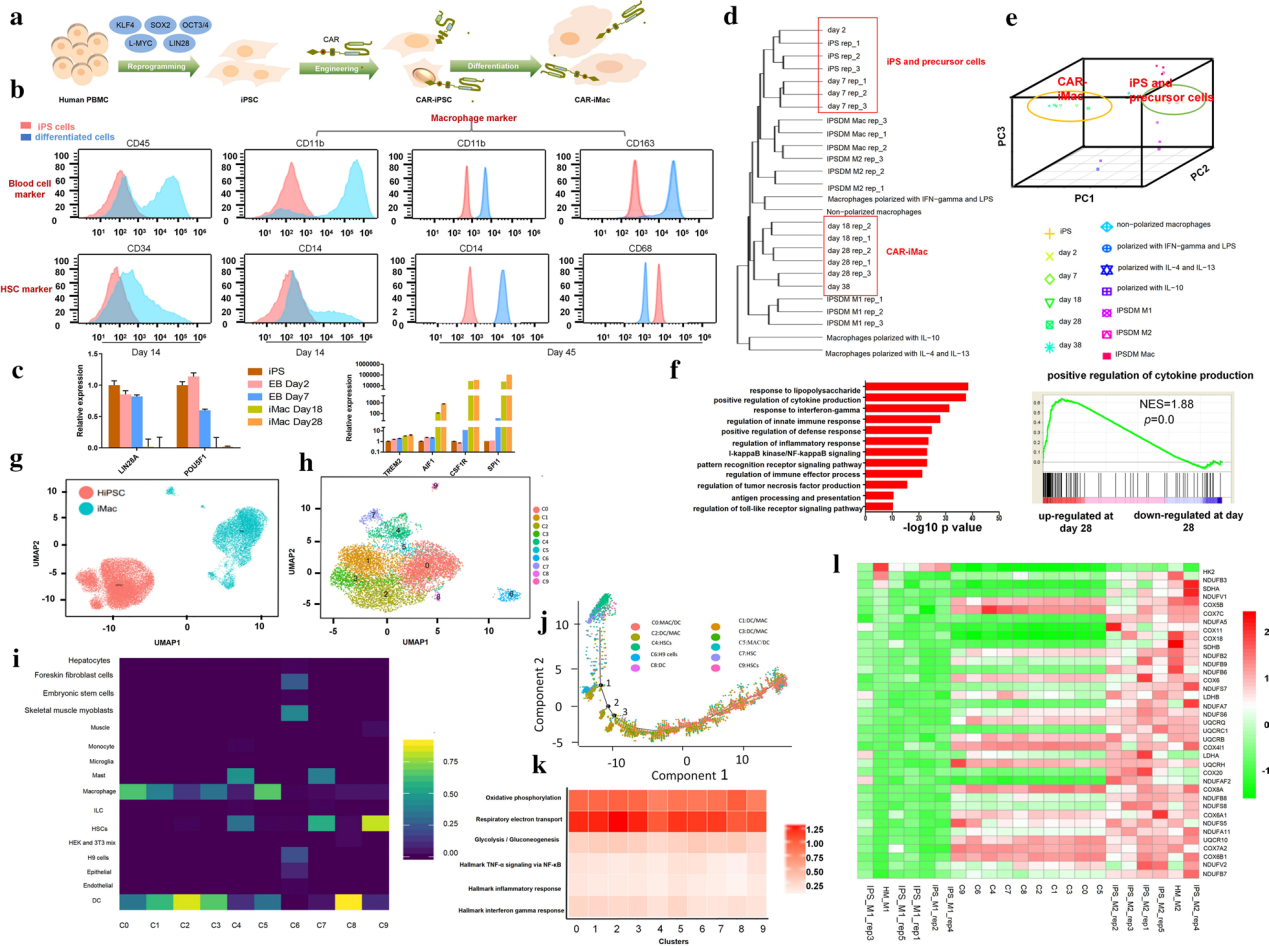
CAR-expressing iPSCs can differentiate into CAR-macrophage cells. **a** Overview of deriving CAR-iMacs from CAR-iPSCs. **b** Flow cytometry analysis of iPSC-derived cells at different stages of differentiation with stage-specific markers. **c** qRT-PCR showing pluripotent marker gene and key macrophage marker gene expression at different stages of CAR-iPSC differentiation. *n* = 3, error bar: standard error of the mean. **d** Hierarchical clustering of transcriptomes of CAR-iPSCs, their differentiated cells, and primary and untransduced iPSC-differentiated macrophages in different states. **e** Principal component analysis (PCA) of the same samples as in **d**. **f** Top GO terms enriched in genes up-regulated on day 28 differentiated CAR-iMac cells compared with CAR-iPSCs, and the right panel is an example of GSEA analysis of the GO terms. *NES* normalized enrichment score. *P* = 0: *P* value is a very small number. **g** UMAP plot showing separation between human iPSCs and CAR-iMac cells. **h** UMAP plot showing subpopulation clustering of CAR-iMac cells. Ten clustered C0–C9 were identified and labeled as 0–9 with different colors. **i** Heatmap showing blasting the C0-C9 clusters of cells illustrated in **g** against a human single-cell atlas database containing single-cell RNA-seq data of hundreds of cell types including macrophages (https://scibet.cancer-pku.cn). **j** Trajectory analysis of differentiated cells along a pseudotime axis. **k** Heatmap showing averaged expression of M1 or M2 signature pathway genes in different clusters of cells illustrated in **i**. **l** Heatmaps to compare (benchmark) the 10 clusters (C0-C9) of CAR-iMac cells against previously published M1 or M2 polarized macrophages using metabolism genes. Human iPS cells differentiated macrophages polarized by IFN-γ and LPS; *IPS_M2:* Human iPS cells differentiated macrophages polarized by IL-4; *HM_M1:* Human PBMC-derived macrophages polarized by IFN-γ and LPS; *HM_M2:* Human PBMC-derived macrophages polarized by IL-4

First, we started from deriving iPSCs from peripheral blood mononuclear cells (PBMC) of a healthy donor with non-integrating episomal vectors encoding reprogramming factors (Fig. 1a), and isolated single iPSC clones (Additional file 1: Fig. S1). The materials and methods are shown in detail in the Additional file 2. Then, we compared different CD19 CARs and chose the conventional CAR to introduce into the iPSCs by lentiviral transduction (Fig. 1a and Additional file 1: Fig. S2a–g). Then, we established a protocol of myeloid/macrophage differentiation to induce CAR-iPSCs toward myeloid cell lineages (Additional file 1: Fig. S2h). Differentiated cells showed typical macrophage marker gene expression (Fig. 1b, Additional file 1: Fig. S4b–i). The products can be collected from 20 to 30 day for multiple times to allow further expansion to have a final yield of above 50-fold of the starting iPSCs (Additional file 1: Fig. S3a), with high purity indicated by ~ 100% of CD11b and CD14 expression at later days (Fig. 1b). Key macrophage marker genes were induced, whereas pluripotent marker genes disappeared (Fig. 1c; Additional file 3: Table S1). RNA-sequencing using differentiated cells showed that iPSCs clustered with precursor cells, and late-day differentiated cells clustered with primary macrophage cells, or untransduced iPSC-differentiated macrophage cells from previous studies [5, 6](Fig. 1d, e). GO analyses showed strong enrichment of innate immunity-related functions in 28-day differentiated cells (Fig. 1f; Additional file 4: Table S2).

Next, we further dissected their subpopulations by performing single-cell RNA-sequencing analysis. These cells clustered away from undifferentiated CAR-iPSCs (Fig. 1g), and they appeared to be largely homogenous with only a small number of cells not clustered with the main population (Fig. 1h). Blasting the differentiated single cells in a database of human cell atlas containing single-cell RNA-sequencing data revealed that these iMac cells mainly clustered with macrophages (Fig. 1i and Additional file 1: Fig. S6a). Trajectory analysis revealed that CAR-iMac cells went through a path from HSC to macrophage and DC cells without major branches (Fig. 1j, Additional file 1: Fig. S5c).

Moreover, all 10 clusters of differentiated cells showed strong signatures of M2 state [7–10] (Fig. 1k, Additional file 1: Fig. S6b). We further compared the single-cell data in the 10 clusters with bulk RNA-seq data from the LPS/IFN-γ-polarized M1 cells or IL-4/IL-10-polarized M2 cells, by examining M1/M2-associated genes (Fig. 1l, Additional file 1: Fig. S5d), and found that most clusters were more similar to the M2 state particularly when using metabolism genes as markers [5, 6, 11–15].

**Fig. 2:**
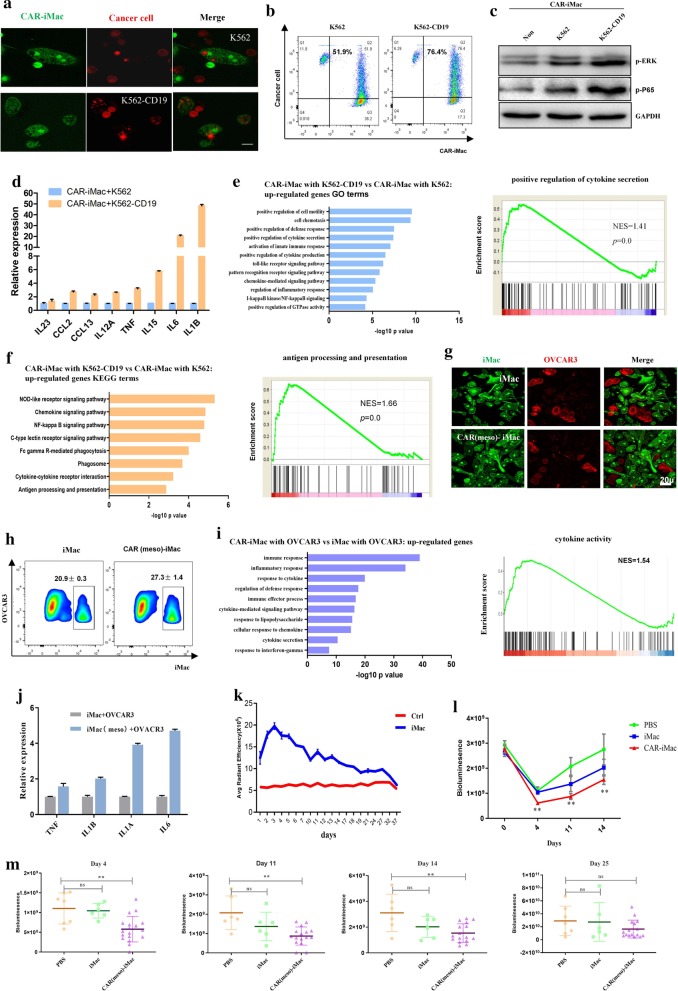
CAR-iMac cells showed antigen-dependent phagocytosis and anticancer cell functions in vitro and in vivo. **a** Confocal microscopy pictures showing phagocytosis of K562 or K562-CD19 cells (red) by CAR (CD19)-iMac cells (green). **b** Flow cytometry showing phagocytosis of K562 or K562-CD19 cells by CAR (CD19)-iMac cells. **c** Western blotting showing phosphorylation of ERK and NF-κB P65 in CAR (CD19)-iMac cells in the indicated conditions. **d** qRT-PCR showing cytokine gene mRNA expression when CAR (CD19)-iMac cells were incubated with K562 or K562-CD19 cancer cells for 24 h. *n* = 3, error bar: standard error of the mean. **e** Top GO terms enriched in genes up-regulated in CAR-iMac cells. Right panel is GSEA analysis of “positive regulation of cytokine production.” **f** Top KEGG pathways enriched in genes up-regulated in CAR-iMac cells. Right panel is GSEA analysis of “antigen processing and presentation.” **g** Confocal microscopic images showing phagocytosis of OVCAR3 cells (red) by iMac or CAR (meso)-iMac cells (green). **h** Flow cytometry showing phagocytosis of OVCAR3 ovarian cancer cells by iMac or CAR (meso)-iMac cells. **i** GO term analysis with RNA-seq data showing the up-regulated genes in CAR (meso)-iMac cells. Right panel is GSEA analysis of “cytokine activity gene.” **j** qRT-PCR showing cytokine gene mRNA expression when iMac or CAR (meso)-iMac cells were incubated with OVCAR3 cells for 24 h. *n* = 3. Error bar: standard error of the mean. **k** 3 × 10^6^ DiR dye-labeled iMac cells were intraperitoneally injected into NSG mice. *n* = 3. Error bars represent standard error of the mean. **l** 4 × 10^5^ of luciferase-expressing ovarian cancer cells (HO8910) were intraperitoneally injected into NSG mice. Mice were treated 4 h later with I.P. injection of PBS, 4 × 10^6^ iMac or 4 × 10^6^ CAR (meso)-iMac cells. Bioluminescence showing tumor development on the indicated days. Statistical analysis was calculated via one-way ANOVA with multiple comparisons between the PBS group and the CAR-iMac group. *P < 0.05; **P < 0.01. **m** Quantification of tumor burden (total flux) by bioluminescent imaging on day 4, 11, 14, and day 25 after CAR-iMac treatment was plotted. Data are presented as the median ± SD, with statistical significance calculated via one-way ANOVA with multiple comparisons. *P < 0.05; **P < 0.01, *ns* not significant

Next, we incubated the CAR (CD19)-iMac cells, CAR (meso)-iMac cells, or control iMac cells with CD19-expressing K562 leukemia cells or mesothelin-expressing OVCAR3/ASPC1 ovarian/pancreatic cancer cells. Compared with K562 alone, K562-CD19 cells were more likely to be phagocytosed by CAR (CD19)-iMacs (Fig. 2a, b), and compared with control cells, CAR (meso)-iMac showed increased phagocytosis activity against OVCAR3 and ASPC1 cells (Fig. 2g, h and Additional file 1: Fig. S7g). Intracellular signaling such as phosphorylation of ERK and NF-κB(P65) proteins were increased in CAR-iMacs co-cultured with CD19-expressing K562 cells compared to K562 cells, or to CAR-iMac cells cultured alone (Fig. 2c). We also examined cytokine gene expression in CAR(CD19)-iMac and CAR (meso)-iMac cells when they were incubated with tumor cells and found antigen-dependent increase in M1 pro-inflammatory cytokine expression(Fig. 2d, j and Additional file 1: Fig. S7h). Moreover, transcriptional analysis showed that CAR(CD19)-iMac cells and CAR(meso)-iMac cells showed strong enrichment of up-regulated genes in GO or KEGG terms of “positive regulation of cytokine secretion,” “antigen processing and presentation,” and “Toll-like receptor signaling pathway,” indicating these cells are more wired toward the pro-inflammatory state, when they encounter the antigen (Fig. 2e, f, i and Additional file 1: Fig. S7i).

When injected into NSG mice, these CAR-iMac cells expanded in vivo till around day 3 for about two fold, and persisted till more than 20 days and gradually disappeared after around 30 days (Fig. 2k). To test their anti-tumor cell activity, we first intraperitoneally injected ovarian cancer cells HO8910 expressing a luciferase gene into NSG mice. In order to further polarize CAR-iMac cells toward M1, we treated them with IFN-γ and washed IFN-γ away before injection (Additional file 1: Fig. S8). CAR(meso)-iMac-treated mice showed reduced tumor burden compared to the control group on day 4, 11, and 14 (Fig. 2l, m). These data demonstrate that the CAR confers anti-cancer cell activities in iMacs in vivo. The efficacy and persistency need to be further improved by designing more effective CARs or further modifying the CAR-iMac cells to stay constitutively active.

## Supplementary information

**Additional file 1.** The main results of differentiation of CAR-expressing iPSCs into CAR-macrophage cells with antigen-dependent phagocytosis and anti-cancer cell functions in vitro and in vivo.

**Additional file 2.** Materials and Methods.

**Additional file 3: Table S1.** Primer sequences for qRT-PCR.

**Additional file 3: Table S2.** RNA-sequencing data analysis of iMac during the differentiation process from iPSCs.

## Data Availability

The datasets used and/or analyzed during the current study are available from the corresponding author on reasonable request.

## Abbreviations

CAR: Chimera antigen receptor
DC: Dendritic cells
ERK: Extracellular-regulated protein kinase
HSC: Hematopoietic stem cell
ICT: Immune-checkpoint therapy
IFN-γ: Interferon gamma
IP: Intraperitoneal injection
iPSC: Induced pluripotent stem cells
LPS: Lipopolysaccharide
MPS: Mononuclear phagocyte system
PBMC: Peripheral blood mononuclear cells
qRT-PCR: Quantitative reverse transcription PCR
