# Supplementary material for: Pluripotent stem cell-derived CAR-macrophage cells with antigen-dependent anti-cancer cell functions
Source: J Hematol Oncol. 2020 Nov 11;13:153. doi: 10.1186/s13045-020-00983-2 (PMC7656711; doi:10.1186/s13045-020-00983-2)
Supplement: Supplementary file 1 — Additional file 1. The main results of differentiation of CAR-expressing iPSCs into CAR-macrophage cells with antigen-dependent phagocytosis and anti-cancer cell functions in vitro and in vivo. [file 13045_2020_983_MOESM1_ESM.docx]

**Additional file 1** Supplementary Figures show differentiation of CAR-expressing iPSCs into CAR-macrophage cells with antigen-dependent phagocytosis and anti-cancer cell functions *in vitro* and *in vivo*

Li Zhang^1,2,^ **^*^**, Lin Tian^1,2,^ **^*^**, Xiaoyang Dai^3,^ **^*^**, Hua Yu^1,^ ^2^**^,*^**, Jiajia Wang^3,^ **^*^**, Anhua Lei^1,2^, Mengmeng Zhu^1,2^, Jianpo Xu^1,2^, Wei Zhao^1,2^, Yuqing Zhu^1,2^, Zhen Sun^1,2^, Hao Zhang^2,4^, Yongxian Hu^2,4^, Yanlin Wang^5^, Yuming Xu^5^, George M. Church^6^ , He Huang^2,4,7, †^, Qinjie Weng^3, †^, Jin Zhang^1, 2, 7,†^

Corresponding authors: Jin Zhang, Qinjie Weng, He Huang

Email: [zhgene@zju.edu.cn](mailto:zhgene@zju.edu.cn), [wengqinjie@zju.edu.cn](mailto:wengqinjie@zju.edu.cn), hehuangyu@126.com

**
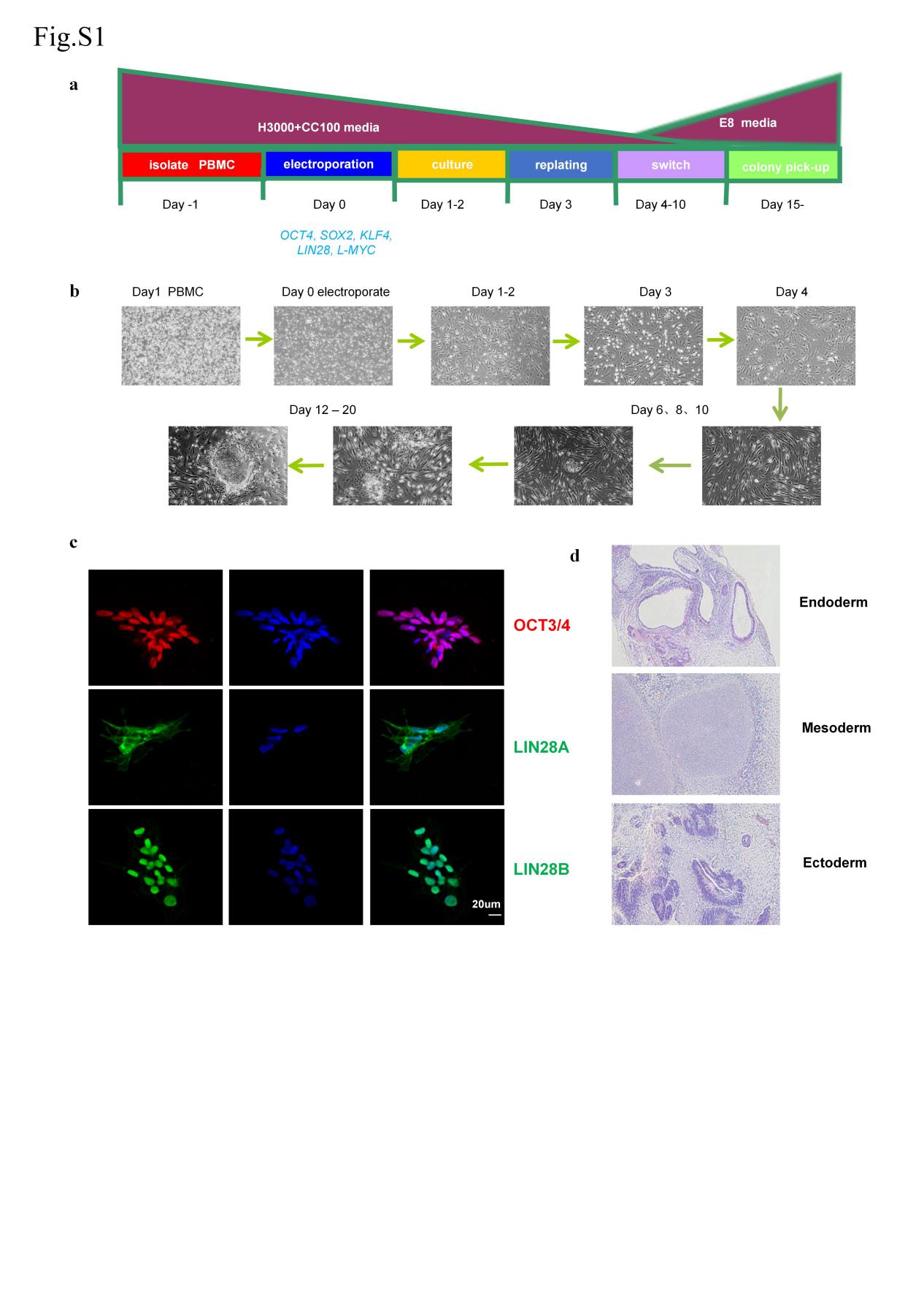
**

Fig. S1 **a** Schematic picture showing procedures of reprogramming from PBMCs to iPSCs. **b** Microscopy images at different stages during reprogramming. **c** Immunostaining showing protein OCT3/4, LIN28A and LIN28B expression in iPSCs. **d** Hematoxylin and eosin staining showing teratomas from transplanted iPSCs including tissues from three germ layers.


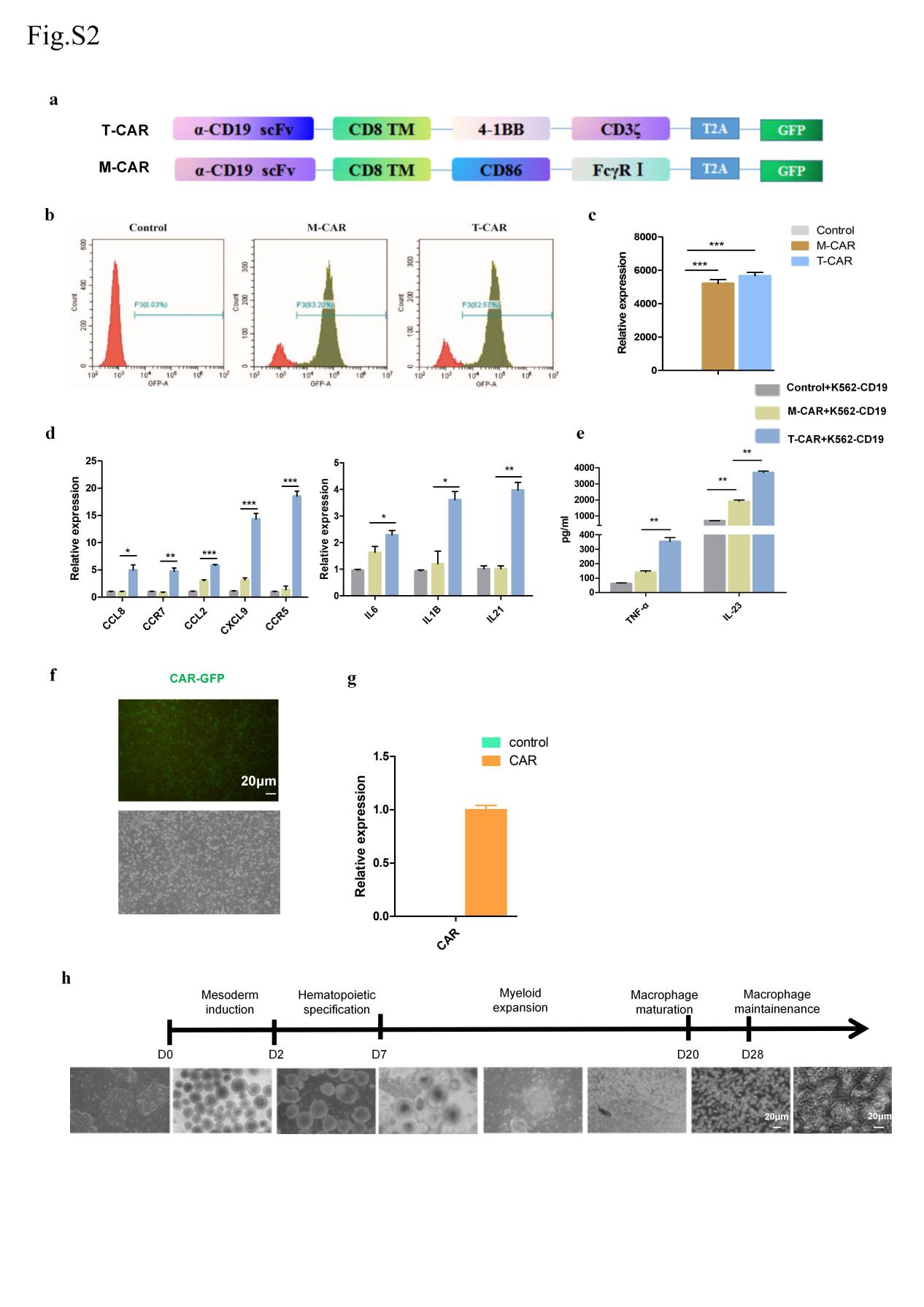


Fig. S2 **a** Schematic illustration of the CD19-T-CAR (the conventional CD19-CAR used in CAR-T cells) and CD19-M-CAR (a modified CAR with domains from macrophage-expressing CD86 and FcγR I) constructs. **b** Flow cytometry analysis of GFP expression in CAR-expressing THP-1 cells and control THP-1 cell without CAR transduction. The levels of expression were comparable in T-CAR-THP-1 and M-CAR-THP-1 cells. **c** qRT-PCR showing CAR expression in T-CAR-THP-1, M-CAR-THP-1 cells and control THP-1 cells. *: *P* < 0.05, **: *P* < 0.01, ***: *P*<0.001, one-way ANOVA. *n*=3, error bar: standard error of the mean. **d** Relative mRNA expression of pro-inflammatory cytokine genes and chemokine genes in the indicated conditions by qRT-PCR. **e** Secreted pro-inflammatory cytokines from the supernatant of co-culture of CAR expressing THP-1 and K562-CD19 cells were detected by ELISA. *: *P* < 0.05, **: *P* < 0.01, ***: *P*<0.001, one-way ANOVA. *n*=3, error bar: standard error of the mean. **f** Microscopy images showing successful transduction of the same T-CAR-GFP as in (**a**) to iPSCs. **g** qRT-PCR showing CAR expression in iPSCs infected with T-CAR lentivirus. Primers were designed against the unique region of anti-CD19 scFv. **h** Overview of the differentiation protocol to derive macrophages from CAR-iPSCs. Representative microscopic pictures showing cells at different stages during differentiation. The last picture is a magnified filed for matured macrophages attached at the bottom of the plate at day 28.


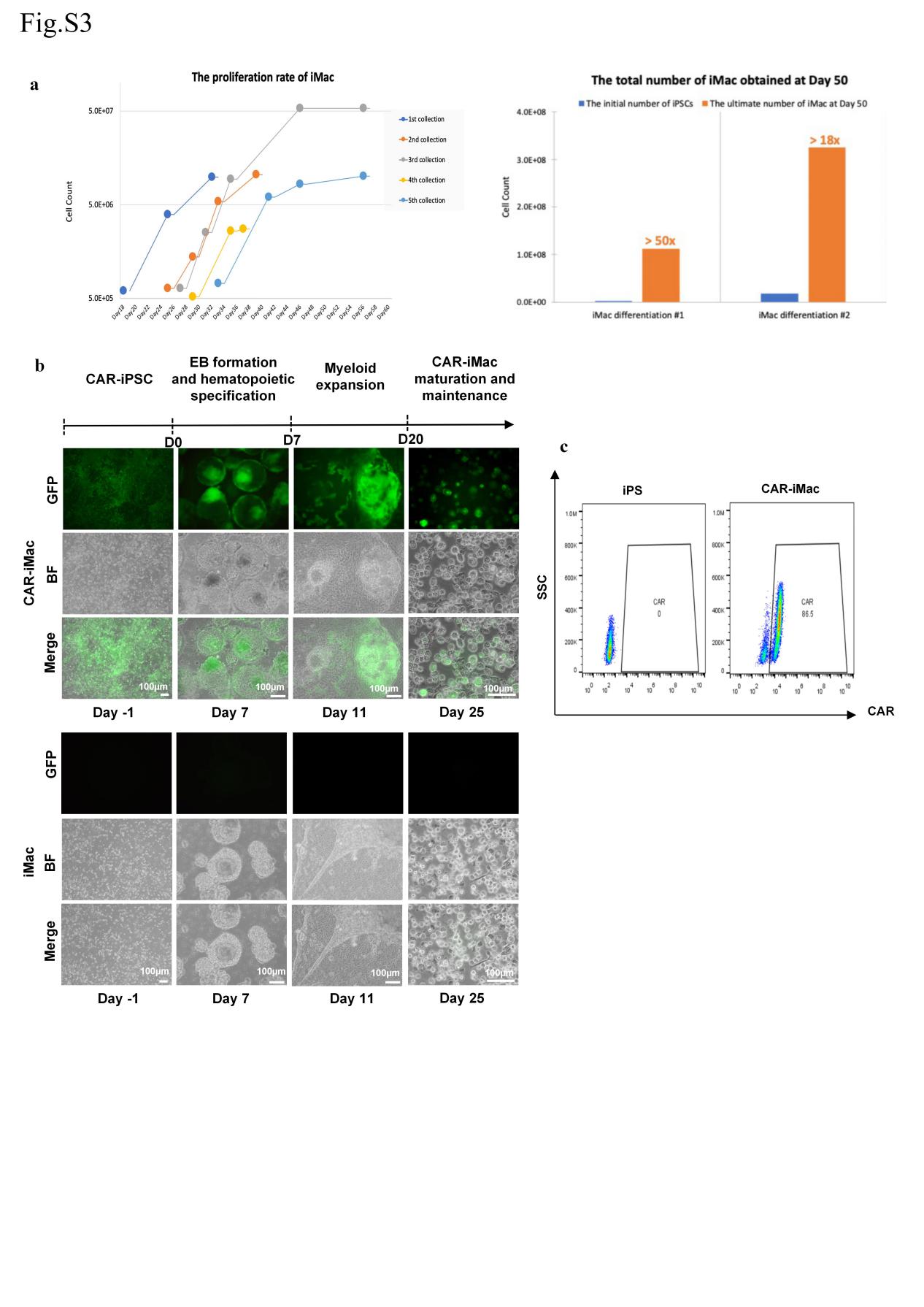


Fig. S3 **a** Differentiation products were collected at Day18 (1^st^ collection), Day 25 (2^nd^ collection), Day 27 (3^rd^ collection), Day 29 (4^th^ collection), and Day 33 (5^th^ collection) to examine the proliferation capacity by trypan blue exclusion method. Right panel, the yield of iMacs from two representative experiments, calculated by adding up product cells from all collections. As some batches were consumed before their proliferation reaches to plateau, the real yield should be more than the calculated yield. **b** Microscopic images of GFP-expressing CAR-iMac cells at different stages of the differentiation process. The CAR and the GFP genes are under the control of the same EF1α promoter. **c** Flow cytometry analysis showing CAR expression on the surface of the CAR-iMac cells.


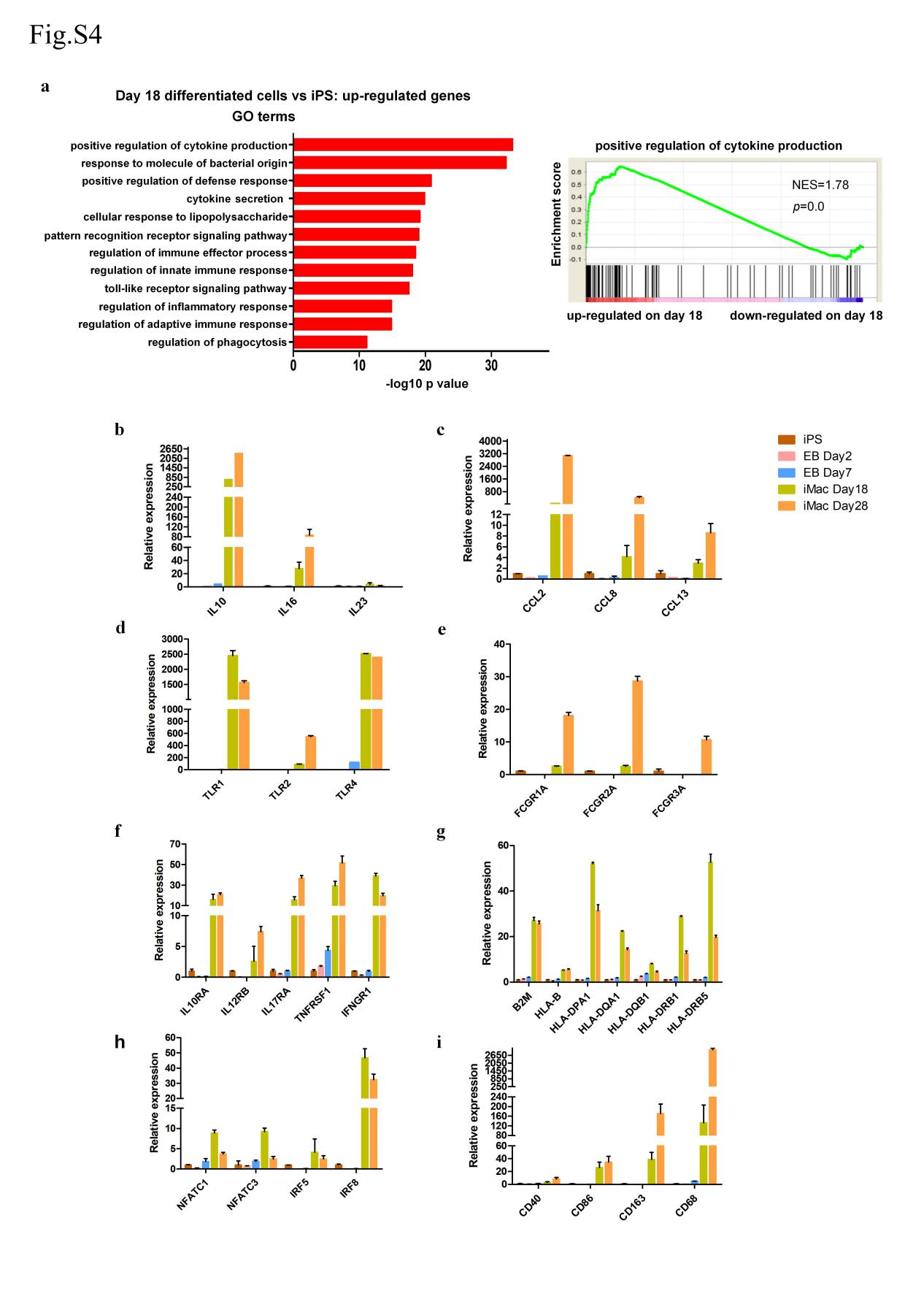


Fig. S4 **a** Top GO terms enriched in genes up-regulated on day 18 differentiated CAR (CD19)-iMac cells compared with CAR-iPSCs. Right panel is an example of GSEA analysis of one GO term “Positive regulation of cytokine production”. NES: normalized enrichment score. *P*=0: p-value is a very small number. **b-i** qRT-PCR showing gene expression of cytokines and chemokines, Toll-like receptors, Fc Receptors, cytokine receptors, HLA genes, immune activation transcription factors, and costimulatory molecules at different days during CAR (CD19)-iMac cell differentiation.


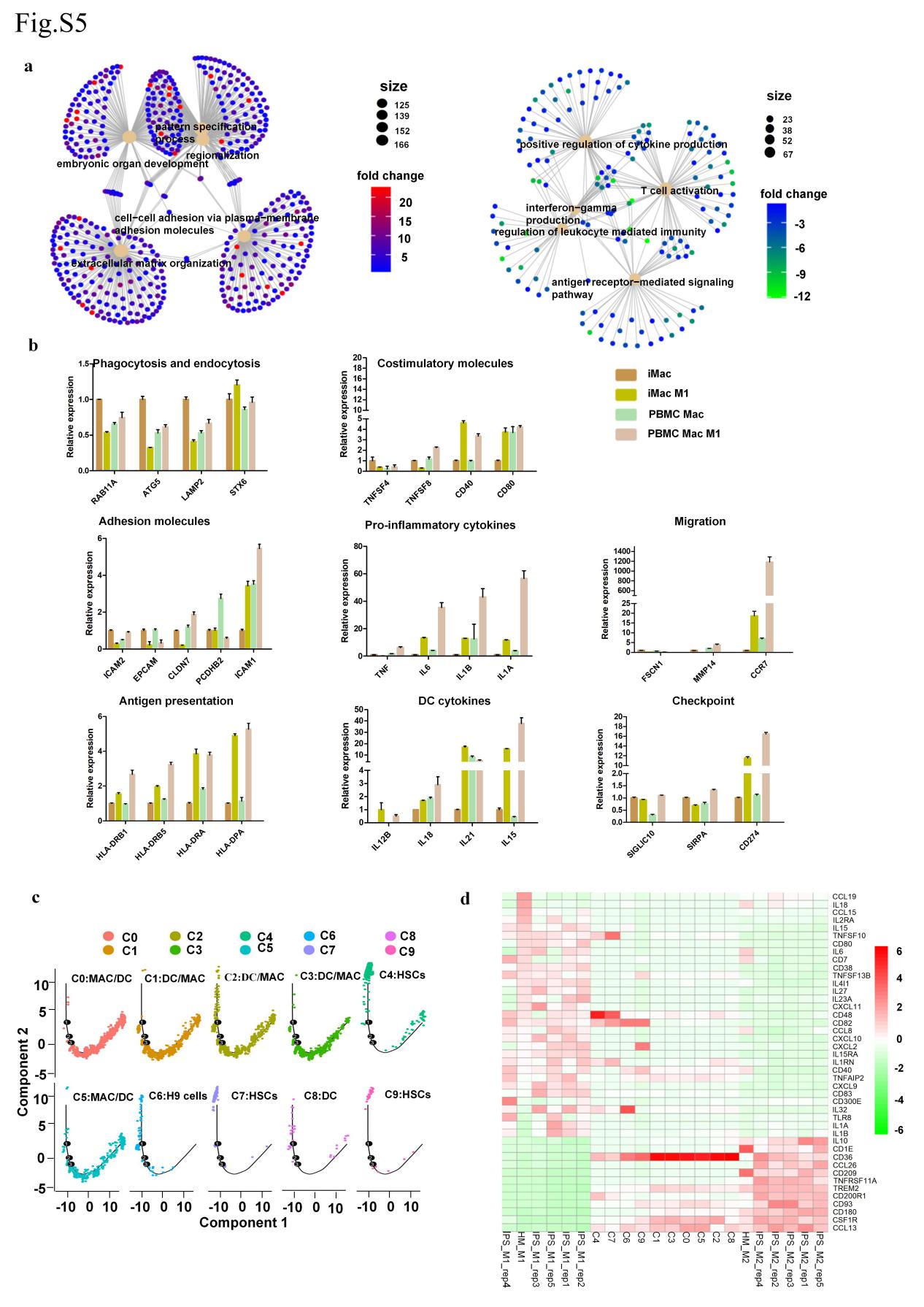


Fig. S5 **a** GO-gene network demonstrating the enriched GO terms and their linked genes; The size of a GO term node is proportional to its P-value in enrichment analysis. The color of a gene node indicates its fold change of expression level between iMac cells and PBMC-derived Mac cells. **b** qRT-PCR showing gene expression representing the indicated functions in iMac cells and PBMC-derived Mac cells in the unpolarized M0 and polarized M1 states. PBMC were cultured in the presence of M-CSF (100 ng/ml) for 7 days, and in the presence of M-CSF + IFN-γ (100 ng/ml) for 24 hours to induce the M1 state. **c** Trajectory analysis of each cluster on the same trajectory along a pseudotime axis. The numbers on the trajectory mean branch points or bifurcating events revealed by the trajectory analysis. MAC/DC means the cluster matches both Macrophage and Dendritic cells. Cluster 6 is not assigned to a specific cell type. **d** Heatmaps to compare (benchmark) the 10 clusters (C0-C9) of CAR (CD19)-iMac cells against previously published M1 or M2 polarized macrophages using top differentially expressed cytokine and chemoattractant genes.


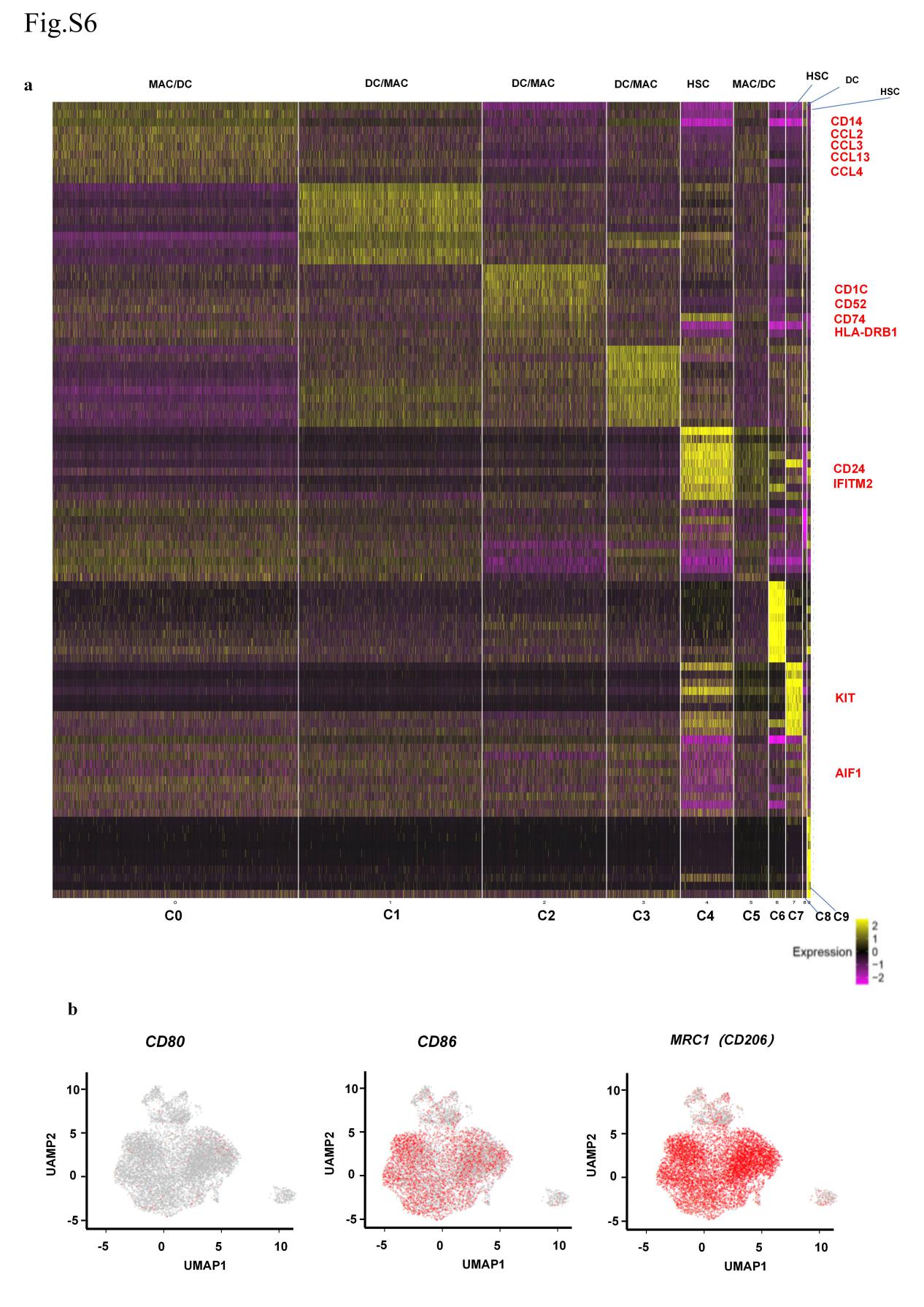


Fig. S6 **a** Heatmap showing gene expression signatures of the 10 clusters and representative genes from single cell RNA-seq analysis of CAR (CD19)-iMac cells**. b** Representative M1 and M2 macrophage marker gene expression in single cell analysis of CAR -iMacs.


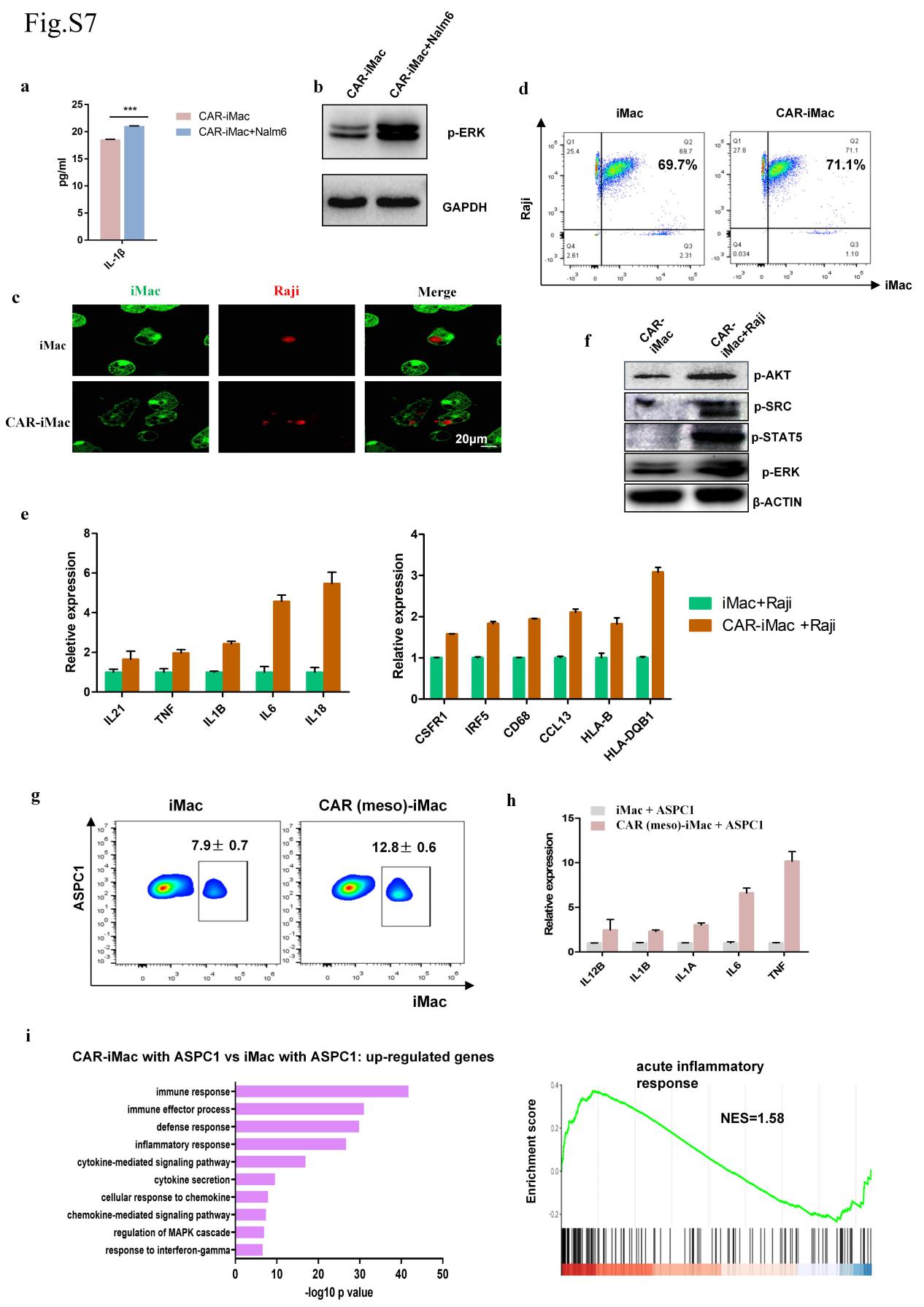


Fig. S7 **a** ELISA showing cytokine secretion in the indicated conditions. ***: *P* < 0.001 *t*-test. *n*=3, error bar: standard error of the mean. **b** Western blotting showing ERK phosphorylation in CAR (CD19)-iMac cells in the indicated conditions. **c** Confocal microscopy pictures showing phagocytosis of Raji cells (red) by CAR-iMac cells (green). **d** Flow cytometry showing phagocytosis of cancer cells by CAR (CD19)-iMac cells. Labeled iMac cells were co-incubated with labeled Raji cells and collected for FACS analysis. Double positive cells represent the iMac cells that engulfed cancer cells. **e** qRT-PCR showing mRNA expression when iMac or CAR(CD19)-iMac cells were incubated with Raji cells. *n*=3, error bar: standard error of the mean. **f** Western blotting showing phosphorylation of AKT, SRC, STAT5 and ERK in iMac cells when stimulated with Raji cells. ACTIN was used as an internal control. **g** Flow cytometry showing phagocytosis of ASPC1 pancreatic cancer cells by iMac or CAR (meso)-iMac cells. *n*=3. **h** qRT-PCR showing cytokine gene mRNA expression when iMac or CAR (meso)-iMac cells were incubated with ASPC1 cells for 24h. Cancer cells were washed off before collecting CAR-iMac. *n*=3, error bar: standard error of the mean. **i** GO term analysis showing the up-regulated genes in CAR (meso)-iMac cells compared with iMac cell when both cells were co-cultured with ASPC1 cells. GSEA analysis of representative acute inflammatory response gene set showing it is up-regulated in CAR (meso)-iMac cells compared with iMac cell when both cells were co-cultured with ASPC1 cells.


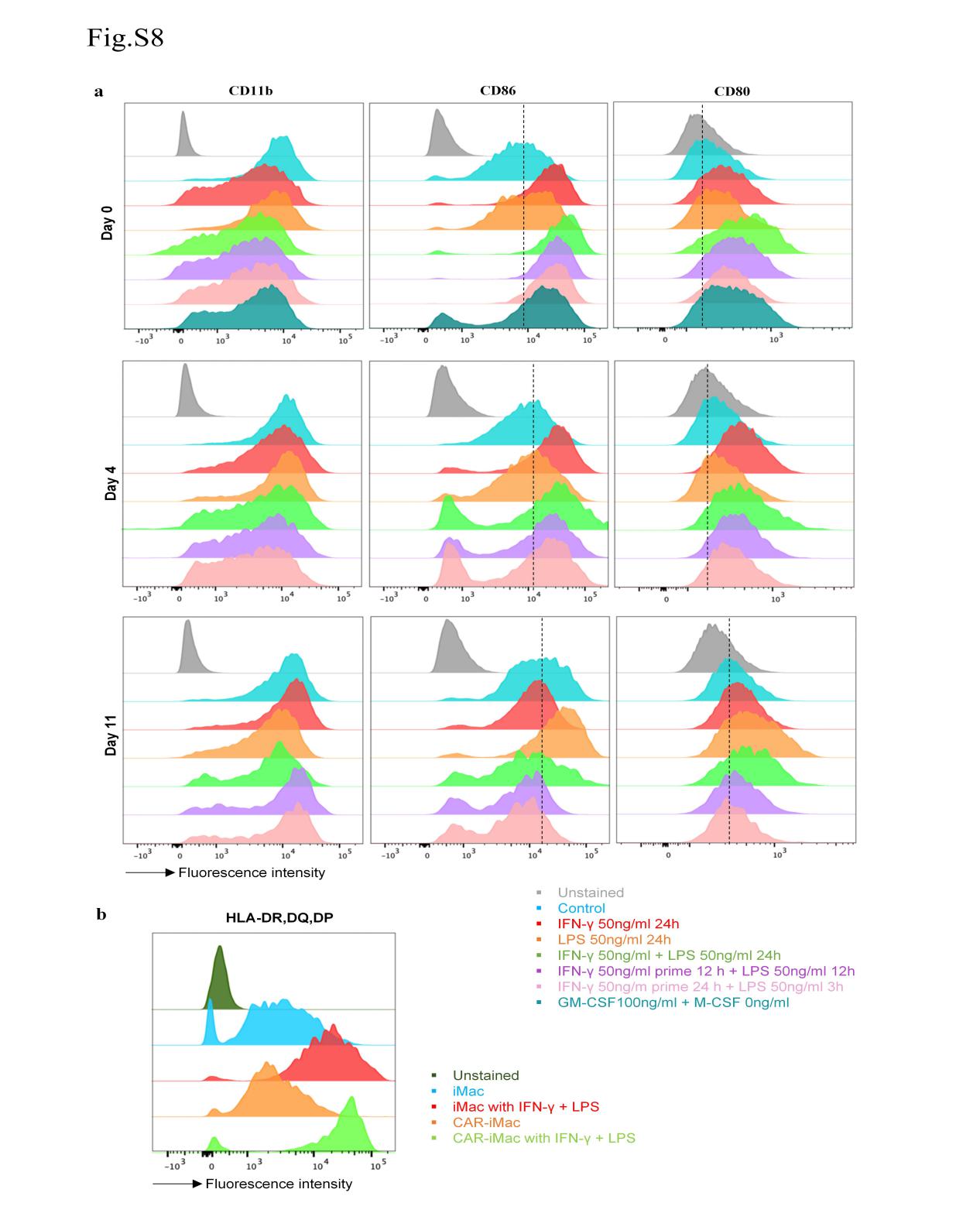


Fig. S8 **a** iMac cells were stimulated by IFN-γ or/and LPS with the indicated concentrations and time. After the simulation, IFN-γ or LPS were washed away by changing fresh medium (10% FBS-containing RPIM-1640 medium with supplemented with 100 ng/ml GM-CSF and 50 ng/ml M-CSF). The CD11b, CD80, and CD86 expression were measured by flow cytometry after 0, 4, and 11 days of stimulation, respectively. For all conditions, iMac cells were cultured in 10% FBS-containing RPIM-1640 medium supplemented with 100 ng/ml GM-CSF and 50 ng/ml M-CSF, except for one condition without M-CSF (jasper color graph at Day 0). **b** Surface expression levels of HLA-DR, DP, DQ on iMac and CAR-iMac treated with (or not) IFN-γ (100 ng/ml) for 24 hours before exposing to LPS (100 ng/ml) for 3 hours.

**
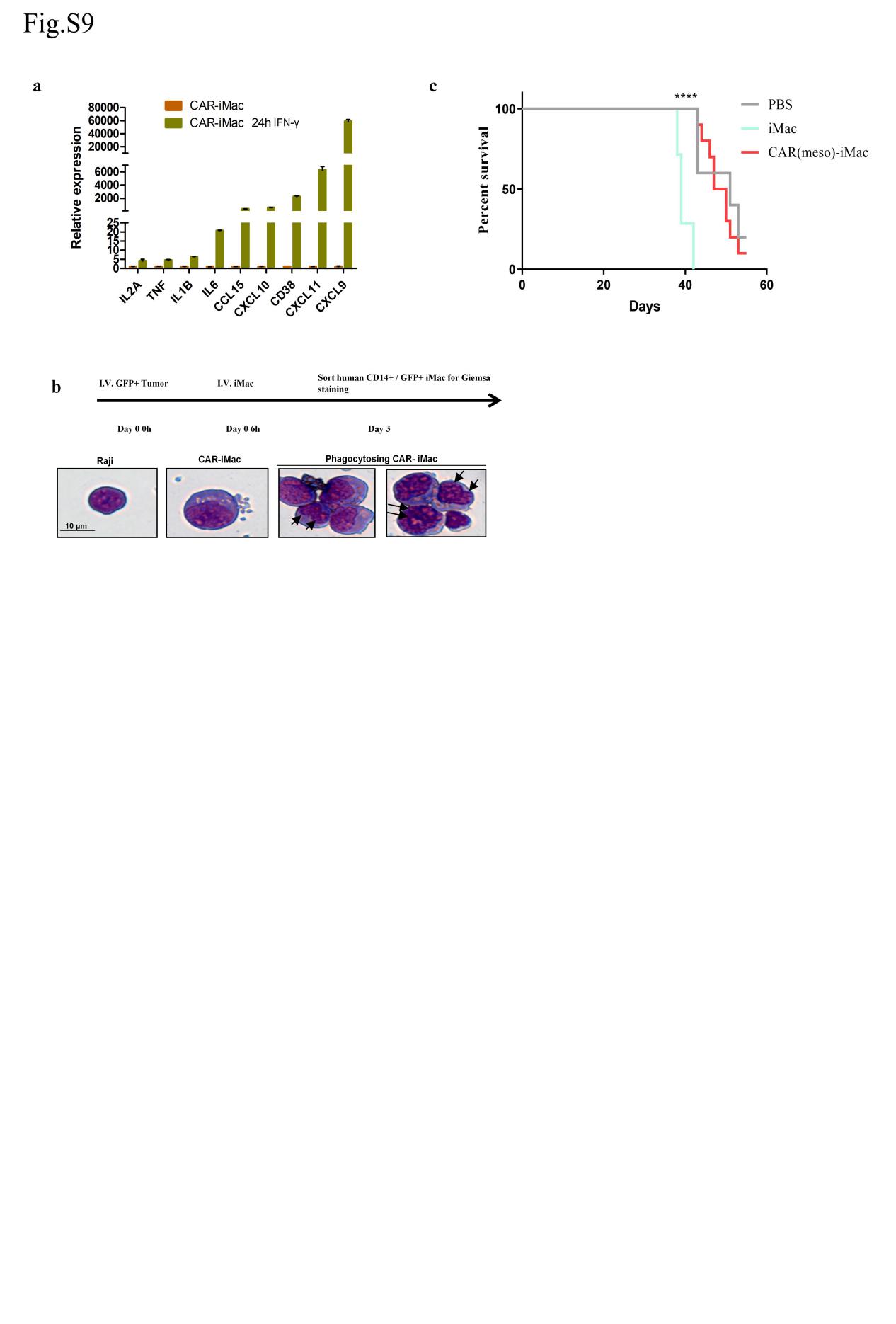
**

Fig. S9 **a** qRT-PCR showing M1 cytokine gene expression in CAR-iMac polarized with IFN-γ (100 ng/ml) for 24h**.** **b** Giemsa staining of FACS-sorted human CD11b and GFP double positive cells showing that CAR (CD19)-iMac cells phagocytosed Raji cells *in vivo.* 4x10^5^ GFP-labeled cancer cells were injected intravenously, and 6 hours later, 1x10^6^ CAR-iMac were injected intravenously. At day 3, cells were sorted for staining. Black arrows point to the multiple nucleus in the isolated macrophages. **c** Kaplan-Meier Survival curve of PBS(n=7), iMacs (n=7) and CAR(meso)-iMacs (n=17)-treated groups. Statistical analysis was calculated via the log-rank Mantel–Cox test (iMac versus CAR-iMac, *P* < 0.0001, df=1).
