# Supplementary material for: Pluripotent stem cell-derived CAR-macrophage cells with antigen-dependent anti-cancer cell functions
Source: J Hematol Oncol. 2020 Nov 11;13:153. doi: 10.1186/s13045-020-00983-2 (PMC7656711; doi:10.1186/s13045-020-00983-2)
Supplement: Supplementary file 2 — Additional file 2. Materials and Methods. [file 13045_2020_983_MOESM2_ESM.docx]

**Additional file 2**

**Materials and Methods**

**Cell culture**

Human iPSCs were cultured in E8 or mTeSR medium (85851, 85852, STEMCELL Technologies) with Matrigel Matrix (354277, Corning) plate. 293T and MEF cells were cultured in high-glucose DMEM (11960-069, Gibco) containing 10% FBS (10099-141, Gibco), 100 µM nonessential amino acids (GNM71450, GENOM) and 100 U/ml penicillin, 100 µg/ml streptomycin (15140-122, Gibco). K562-CD19 cells expressing CD19 were prepared by lentiviral transduction of K562 cells, followed by FACS sorting of CD19^high^ cells. THP-1, Raji, Nalm6 and K562 cells were maintained in RPMI1640 (SH30809, HyClone) medium, containing 10% FBS (10099-141, Gibco), 100 µM nonessential amino acids (GNM71450, GENOM) and 100 U/ml penicillin, 100 µg/ml streptomycin (15140-122, Gibco).

**Reprogramming**

Peripheral blood mononuclear cells (PBMC) were collected from a volunteer donor after informed consent was obtained. PBMCs were cultured in StemSpan^TM^ H3000 (009850, STEMCELL Technologies) + StemSpan™ CC100 (02690, STEMCELL Technologies) medium on day -1. 10^6^ PBMCs were transfected with Yamanaka episomal reprogramming plasmids (pCXLE-hSK 1 μg, pCXLE-hUL 1 μg and pCXLE-hOCT3/4 1 μg) by Lonza 4D-Nucleofector using the P3 Primary Cell 4D Nucleofector X Kit (V4XP-3024) and the FI115 program (Lonza). Electroporated cells were cultured in RPMI1640 (SH30809, HyClone) medium containing 10% FBS (10099-141, Gibco), followed by changing to H3000+CC100 medium 4 hours later. All cells were transferred to a new MEF feeder plate three days after electroporation in H3000+CC100 medium. 200 μl E8 medium (#05990, STEMCELL Technologies) were added the next day. On day 6, day 8 and day 10, 1 ml of medium was collected, centrifuged at 250 rcf for 5 min, then the pellet was resuspended in 1.2 ml of E8 medium. On day 11, the medium was completely changed to E8, and was then changed daily. iPSC colonies appeared at ~15–25 days after electroporation. iPSC lines were maintained in culture on Matrigel Matrix (354277, Corning) plates with E8 medium and passaged every 3-5 day.

**CAR macrophage cells production**

We designated T-CAR and M-CAR according to the intracellular domains of T cell receptor CD3ζ subunit and macrophage FcγRI receptor. They are composed of the following components from 5*'* to 3*'* : anti-CD19 scFv, the transmembrane region of human CD8α (amino acids 183-206), and the cytoplasmic portion of human 4-1BB (amino acids 214-255) plus the cytoplasmic part of the human CD3ζ (amino acids 52-164) for T-CAR, or the cytoplasmic portion of the human CD86 (amino acids 269–329) plus the cytoplasmic part of the human FcγRI (amino acids 314–374) for macrophage-specific CAR (M-CAR). T-CAR and M-CAR gene fragment sequences were synthesized by GenScript Biotech. The synthesized sequences were cloned into the EcoRI and XbaI sites of Lenti-EF1A-CD19-CAR-T2A-EGFP-Puro vector using restriction enzyme cloning and ligation. Lentivirus was produced by HEK293T cells cultured in 10 cm dishes (Corning) transfected with pMD2.G, psPAX2 and the lentiviral backbone vector containing the constructs of interest using Lipofectamine 2000 (Invitrogen). The medium was replaced with fresh medium 6 hours post transfection to remove transfection reagent. Lentivirus-containing medium was collected after 24 hours and 48 hours, filtered with a 0.22 μm filter (Millipore), and mixed with half volume of 25% PEG6000 overnight at 4˚C. The next day, medium was concentrated at 4000 rpm for 20 min at 4˚C. The supernatant was discarded, and the pellet was resuspended with PBS. The concentrated virus was added to iPSCs and cells were treated with puromycin (0.25 μg/ml) 48 hours post infection for 3 days to select successfully transduced clones. Then, we used the protocol of macrophage differentiation to produce CAR-iMac. Also, CAR-expression THP-1 cells were generated with lentivirus transduction. Briefly, 1~2×10^6^ THP-1 cells were transduced with lentivirus-M-CAR or T-CAR (MOI=20) with polybrene (0.5 μg /ml) by centrifugation at 800×g for 30 min, and subsequently, transduced THP-1 cells were cultured and selected with puromycin (1 µg/ml) for 7 days. After 7 days, cells were analyzed with flow cytometry for GFP expression to determine CAR expression.

**Macrophage differentiation from CAR-iPSCs**

Undifferentiated CAR-iPSCs were treated with Versene (15040-066, Gibco) for 6 min and transferred to low-attachment plates (3471, Corning) to allow formation of embryoid bodies (EBs) in mTeSR medium (85850, STEMCELL Technologies). Formation of embryoid bodies was facilitated by an overnight incubation at 37 °C in 5% CO2 with shaking at 50 rpm. Primitive streak-like mesodermal progenitor cells were generated by incubation of EBs with BMP-4 (10 ng/ml) and bFGF (5 ng/ml) in APEL II medium (05270, STEMCELL Technologies) (day 1). Then, hematopoietic specification was achieved in the presence of BMP-4 (10 ng/ml), bFGF (5 ng/ml), VEGF (50 ng/ml) and SCF (100 ng/mL) in APEL II medium (day 2-7). Myeloid lineage differentiation was achieved by supplementing the medium with bFGF (10 ng/ml), VEGF (50 ng/ml), SCF (50 ng/mL), IGF-1 (10 ng/mL), IL-3 (25 ng/mL), M-CSF (50 ng/mL) and GM-CSF (50 ng/mL) in APEL II medium (day 8-9). On day 10, plate 40-50 EBs onto 1 well of 6-well plate pre-coated with Matrigel Matrix (354277, Corning) and switch medium to StemPro™-34 SFM medium (10639011, Gibco) with bFGF (5 ng/ml), VEGF (50 ng/ml), SCF (50 ng/mL), IGF-1 (10 ng/mL), IL-3 (25 ng/mL), M-CSF (50 ng/mL) and GM-CSF (50 ng/mL) (day 10-20). Floating cells from the wells were collected and replated onto new empty Matrigel plates, in myeloid maturation medium (MM medium) containing bFGF (5 ng/ml), VEGF (50 ng/ml), SCF (50 ng/mL), IGF-1 (10 ng/mL), IL-3 (25 ng/mL), M-CSF (100 ng/mL) and GM-CSF (100 ng/mL) for 2 days. From day 22 to day 27, cells were cultured with MM medium without IL-3. Successfully differentiated iPSC-macrophages were maintained in RPMI1640 medium with 10% FBS or H3000 medium containing M-CSF (100 ng/mL) and GM-CSF (100 ng/mL) from day 28. All recombinant factors were purchased from PeproTech.

***In vitro* phagocytosis assay**

Tumor cells of K562, Raji, OVCAR3 or ASPC1 were lively labeled by CellTracker Deep Red Dye (C34565, Thermo Fisher), and co-cultured with iPSC differentiated macrophage cells lively labeled by CellTracker CMFDA Dye (C7025, Thermo Fisher) in a 1:5 ratio. After 24 hours, the cells were washed by PBS, treated and lifted by TrypLE Selected Enzyme, and analyzed by flow cytometry. For immunofluorescence, labeled cells were seeded onto glass bottom cell culture plate (801002, NEST) for 24 hours before microscopy.

**Flow cytometry analysis**

Cells were harvested and cell suspensions were pelleted and washed with 0.1% BSA in PBS, then stained with antibodies for 10 minutes at room temperature. Flow cytometry antibodies: CD11b-FITC (301330, BioLegend), CD163-PE (333606, BioLegend), CD86-FITC (374204, BioLegend), CD80-FITC (305205, BioLegend), HLA-DR, DP, DQ-PE (361715, BioLegend). All flow cytometry experiments were done on BD Fortessa and analyzed on FlowJo software.

**RNA extraction, cDNA** **and real-time PCR**

Total RNA was extracted from human iPSCs and various stages of differentiated cells using RNA Isolation Mini kit (RC101-01, Vazyme) according to the manufacturer’s protocol, and 1 μg RNA was reverse transcribed to cDNA with HiScript II Q RT Super Mix (R223-01, Vazyme). Gene expression was analyzed with SYBR-Green qPCR Master mix. Primer sequences are listed in Additional file 3：Table S1.

**RNA-sequencing library preparation and sequencing**

A total amount of 2 μg RNA per sample was used as input materials for the RNA sample preparation. mRNA was purified from total RNA using poly-T oligo-attached magnetic beads. Purified mRNA was fragmented at 94°C for 15 minutes by using divalent cations under elevated temperature in NEBNext first strand synthesis reaction buffer (5X). First strand cDNA was synthesized using random primer and ProtoScript II reverse transcriptase in a preheated thermal cycler as follows: 10 minutes at 25°C; 15 minutes at 42°C; 15 minutes at 70°C. Immediately, second strand synthesis reaction was performed by using second strand synthesis reaction buffer (10X) and the enzyme mix at 16°C for 1 hour. The library fragments were purified with QIAquick PCR kits and eluted with EB buffer, followed by terminal repair, A-tailing and addition of adapters. The products were retrieved and PCR was performed for library enrichment. The libraries were sequenced on an Illumina NextSeq platform.

**RNA-sequencing data analysis**

All bulk RNA-seq reads were trimmed using Trimmomatic (Version 0.36) software with the following parameters “ILLUMINACLIP: TruSeq3-PE.fa:2:30:10 LEADING:3 TRAILING:3 SLIDINGWINDOW:4:15 MINLEN:36” and were further quality-filtered using fastq_quality_trimmer command contained in FASTX Toolkit (Version 0.0.13, http://hannonlab.cshl.edu/fastx_toolkit/) with the minimum quality score 20 and minimum percent of 80% bases that has a quality score larger than this cutoff value. The high-quality reads were mapped to the GRCh38 genome by HISAT2, a fast and sensitive spliced alignment program for mapping RNA-seq reads, with -dta parameter. PCR duplicate reads were removed using Picard tools and only uniquely mapped reads were kept for further analysis. The expression levels of genes were calculated by StringTie (Version v1.3.4d, with -e -B -G parameters) using Release 28 (GRCh38.p12) gene annotations downloaded from GENCODE data portal. To obtain reliable and cross-sample comparable expression abundance estimation for each gene, reads mapped to reference genome were counted as TPM (Transcripts Per Million reads) based on their genome locations. Differential expression analysis of genes in different samples was performed by DESeq2 package using reads count matrix produced from a python script “prepDE.py” downloaded from StringTie website (http://ccb.jhu.edu/software/stringtie/). The clustering and PCA analysis were conducted using “pheatmap” package and “prcomp” function of R software, respectively. Based on the curated human pathway annotations downloaded from KEGG database, the GSEA software (http://software.broadinstitute.org/gsea/index.jsp) was used to perform gene set enrichment analysis. Processed gene expression data are listed in Additional file 4：Table S2.

**Generation of single cell GEMs and sequencing libraries**

10000 cells (90%–95% viability) were captured per sample in a 10X Chromium device using 10X V2 Single Cell 3*'* Solution reagents (10X Genomics, Inc) at a concentration of 1000 cells/μl. Experiment was performed according to manual instructions. After the GEM-RT incubation, barcoded-cDNA was purified with DynaBeads cleanup mix, followed by 10-cycles of PCR amplification (98°C for 3 min; [98°C for 15 s, 67°C for 20 s, 72°C for 1 min] x 10; 72°C for 1 min). The total cDNA of single-cell transcriptomes was fragmented, double-size selected with SPRI beads (Beckman), followed by 12 cycles sample index PCR amplification (98°C for 45 s; [98°C for 20 s, 54°C for 30 s, 72°C for 1 min] x 10; 72°C for 1 min), then another double-size selection with SPRI beads was performed before sequencing (Illumina NextSeq platform).

**Single cell RNA-sequencing data analysis**

The 10X single-cell gene expression analysis pipeline was used to produce feature-barcode gene expression matrix. The obtained single-cell gene expression matrix was analyzed with Seurat v2.3.4 (https://satijalab.org/seurat/). We excluded the genes with expressed cell number smaller than 3 and the cells with nUMIs smaller than 500 or the expression percentages of mitochondrial genes larger than 0.2 and used 16 Principle Components (PCs) for UMAP and clustering analysis. The cell identities (types) of the produced single-cell sequencing data were predicted by an entropy-based predictor (<http://scibet.cancer-pku.cn/>). All other data analyses and visualization were performed in R/Bioconductor utilizing custom R scripts. Constructing single-cell trajectory (pseudotime ordering) was done by Monocle v2.4 R package on the basis of the transcriptome profiles using default settings[1].

**Immunofluorescence staining**

Human iPSCs were plated on Matrigel Matrix covered glass coverslips. Cells were fixed with 4% PFA for 20 min followed by permeabilization with 0.5% Triton X-100/PBS for 10 min at RT. After washing cells with 0.1% Triton X-100/PBS for three times, cells were blocked in blocking buffer (2% BSA, 0.1% Triton X-100/PBS) for 20 min and stained with a primary antibody overnight. After washing three times for 5 min with 0.1% Triton X-100/PBS, cells were stained with a secondary antibody for 1 h at 37 ˚C. Antibodies used: For LIN28A and LIN28B (Cell Signaling Technology), rabbit antibodies at 1:300 and a donkey anti-rabbit Alexa Fluor 488-conjugated secondary antibody (Abcam) at 1:400 were used. For OCT3/4 (Santa Cruz), mouse antibodies at 1:200 and a DyLight 594 goat anti-mouse secondary antibody (EarthOx) at 1:400 were used. Following washing for another three times with 0.1% Triton X-100/PBS, DAPI was used for nucleus staining. Cells were then imaged using Zeiss LSM880 fluorescence microscope with a 40× oil objective.

**Analysis of co-cultured supernatant cytokine concentrations**

The cytokine concentrations of human IL-12, IL-23, TNF-α and IL-6 were determined from the co-cultured supernatant of macrophages (CAR-iMac, iMac or THP-1) with tumor cells (K562, CD19-expressing K562, or Nalm6). It was measured using an enzyme-linked immunosorbent assay (ELISA). The following kits were used for quantification of the cytokines: Human IL-6 (E-EL-H0102c), Human IL-12 (E-EL-H0150c), Human IL-23 (E-EL-H0107c) and Human TNF-α (E-EL-H0109c) from Elabscience. All experiments were performed according to the manufacturer’s instructions.

**Teratoma formation assay**

iPSCs were harvested by Gentle Cell Dissociation Reagent at 37 °C for 5 min. Cells (2-3×10^6^) were suspended in 100 μL Matrigel (Corning, 354277). Cells were then injected into dorsal root of the thigh of the immune-deficient mice (NOD/SCID). Teratomas usually formed within 6-8 weeks. Upon removal, the teratomas were fixed, embedded in paraffin, and stained with hematoxylin and eosin.

### *In vivo* anti-cancer cell activity and phagocytosis assays

### NOD-PrkdcscidIl2rgem1/Smoc (M-NSG) mice were obtained from Shanghai Model Organisms Center, Inc. Six- to eight-week-old mice were bred, treated, and maintained under pathogen-free conditions in-house under the Zhejiang University Institutional Animal Care and Use Committee–approved protocols. The anti-tumor activity of CAR-iMac was tested in vivo using two models. In the first model, mice were engrafted with 4×10^5^ of luciferase-expressing leukemia cells (Nalm6) intraperitoneally before treatment. Four days post engraftment, mice were intraperitoneally injected with PBS or 1.2×10^7^ CAR-iMac cells. In the second model, 4×10^5^ of luciferase-expressing ovarian cancer cells (HO8910) were infused via IP. Mice were treated 4 hours later with IP injection of PBS, 4×10^6^ iMac or 4×10^6^ CAR-iMac cells. iMac and CAR-iMac cells were pre-treated with IFN-γ (100 ng/ml) for 24 hours and then LPS (100 ng/ml) for 4 hours before injection. Anti-cancer cell effect was monitored using a small animal imaging system (In-vivo Xtreme) 10-12 min after intraperitoneal injection of 0.2 ml D-luciferin. For *in vivo* iMac survival assays, cells were labeled with a near-infrared fluorescence 1,1-dioctadecyl-3,3,3,3-tetramethylindotricarbocyanine iodide (DiR) dye at 5 μM for 20 min at 37 degree before injected to NSG mice, and the mice were then monitored by a IVIS Lumina series III small animal imaging system for about a month. For *in vivo* phagocytosis assays, blood cells were harvested on day 3 after CAR-iMac injection, and sorted for human CD11b and GFP double positive cells, and those cells were stained with Giemsa staining to observe the phagocytosis effect of CAR-iMac.

**References:**

1. Trapnell C, Cacchiarelli D, Grimsby J, Pokharel P, Li S, Morse M, et al. The dynamics and regulators of cell fate decisions are revealed by pseudotemporal ordering of single cells. Nat Biotechnol. 2014;32(4):381-6.
